# Genetic algorithm for the location control of femtosecond laser filament

**DOI:** 10.1038/s41598-020-69918-8

**Published:** 2020-07-30

**Authors:** Zhi Zhang, Olga Kosareva, Nan Zhang, Lie Lin, Weiwei Liu

**Affiliations:** 1 Institute of Modern Optics, Nankai University, Tianjin Key Laboratory of Microscale Optical Information Science and Technology, Tianjin, 300350 China; 2 International Laser Center, Lomonosov Moscow State University, Moscow, Russia 119991

**Keywords:** Optics and photonics, Applied optics, Lasers, LEDs and light sources, Optical physics, Optical techniques

## Abstract

An adaptive method based on the genetic algorithm (GA) is proposed to control the location of femtosecond laser filament. To verify the feasibility of this method, the simulation results obtained through the GA method are compared with those by the chirp method when femtosecond laser pulses with different pulse energies are used. It is found that the intensity profile and the phase of the femtosecond laser pulses obtained by the GA method are nearly identical to those obtained by the chirp method. It demonstrates that the GA adaptive control method can accurately control the position of the starting point of the filament in the femtosecond laser filamentation.

## Introduction

Since a series of interesting nonlinear processes such as self-focusing, photoionization, intensity clamping, self-phase modulation, self-steepening etc.^1–5^. occur during the femtosecond laser filamentation, the optical filament has attracted wide interests in the field of ultrafast nonlinear optics both theoretically^6–9^ and practically^10–12^. Based on its unique properties, femtosecond laser filamentation has been used in many applications including remote sensing^13–16^, microwave-guiding^17^, lightning discharge control^18^, and artificial precipitation^19^. However, challenges still exist in producing stable and controllable femtosecond laser filaments.

The location control of the femtosecond laser filament is necessary for many applications, especially the remote pollutant detection^20^. Based on the pulse shaping techniques^21,22^, there are many methods that have been developed to control the location of the femtosecond laser filament^23–25^. The chirp method can introduce a quadratic temporal phase to laser pulses by the dispersion compensating elements like prisms, chirped mirrors and gratings. The spatial phase modulation methods can introduce different phases for different spectral components by using spatial light modulator (SLM), deformable mirror, and acousto-optic modulator. All these techniques can achieve the filament location control by manipulating the phase of the laser pulses and influencing the complex nonlinear processes during the filamentation. For example, by modulating the initial phase using the chirp method, Golubtsov et al. have moved the location of the filament, extended the length of the filament, and enhanced the supercontinuum spectrum^24^. By manipulating the spectral phase of the pulses using a SLM, Heck et al. have achieved a specified control for filament position and length in an aqueous solution^26^.

However, in practice the chirp method mainly introduces the linear chirp into the pulse phase which cannot exactly control the complicated dynamics of the femtosecond laser filamentation^21,22^. Therefore, the capability of controlling the filament location is limited for the chirp method. In the frequency domain, when the programmable SLM combined with an optical 4-*f* system^27^ and certain optimization algorithms such as simulated annealing algorithm^28^, particle swarm optimization^29^ and genetic algorithm^30^ are employed to control the filament location, the adaptive and simultaneous multi-parameter control of the filament can be achieved.

In this paper, we investigate the feasibility of using the genetic algorithm (GA) to control the location of the femtosecond laser filament. In the GA method proposed here, the random phase parameters evolve through the repeating operations of selection, crossover, and variation to achieve the filamentation at the target distance. The location of the simulated starting point of the filament based on the GA method and the corresponding calculated pulse intensity and phase are in good agreement with those obtained by the chirp method. Therefore, the proposed GA method with the self-adaptive property is proved to be an effective method to pre-determine the laser phase distribution for generating the optical filament at the desired location.

## Methods

### Chirp method

**Figure 1:**
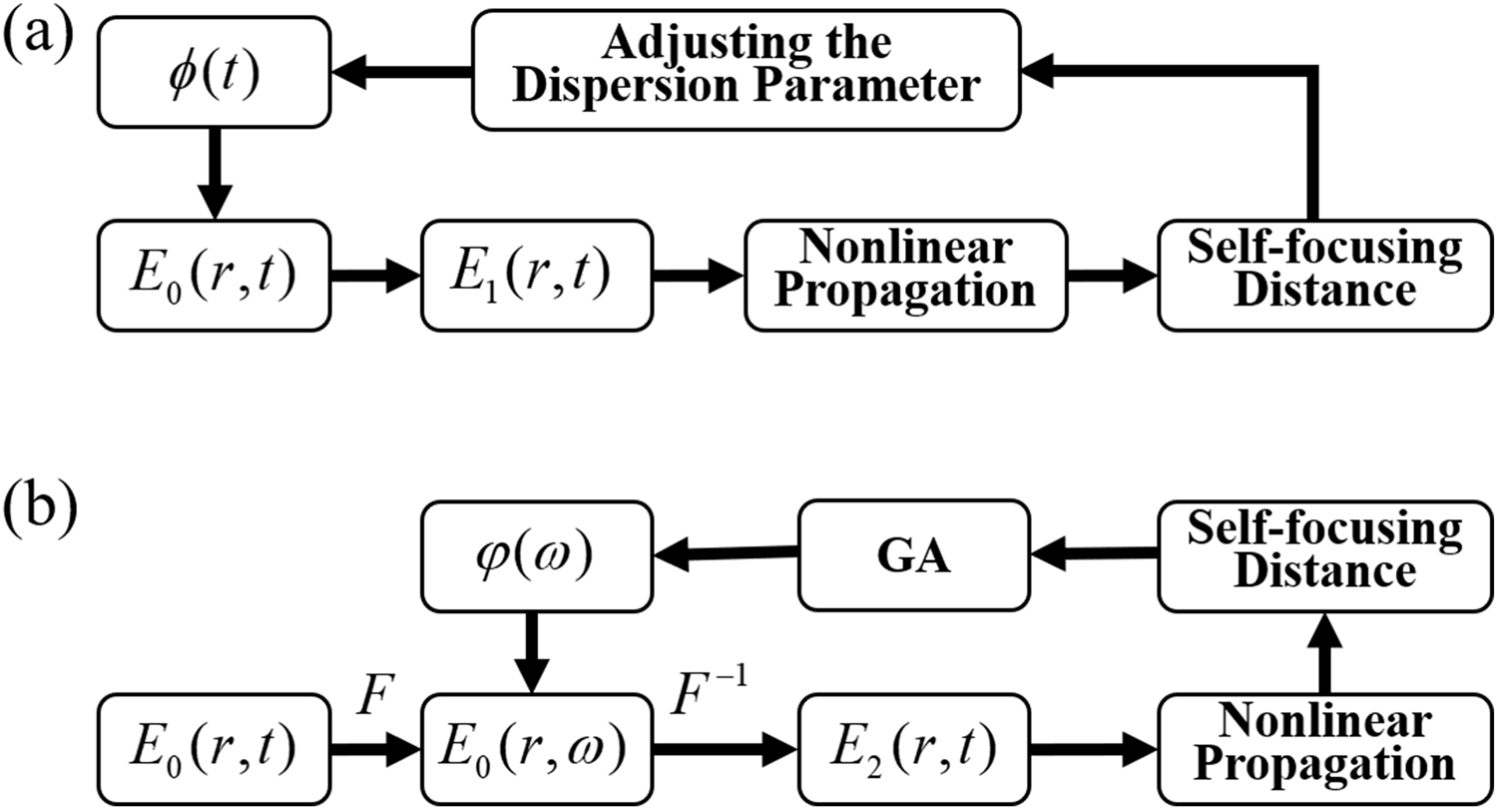
Numerical simulation flow charts for the chirp method (**a**) and the GA method (**b**) respectively. *F* and *F*^−1^ means the forward and inversed Fourier transforms, respectively.

The simulation flow chart for the chirp method is shown in Fig. 1a. *E*_0_(*r*, *t*) represents the chirp-free Gaussian femtosecond laser pulse which can be expressed as:

1 $$ E_{0} (r,t) = (2P_{0} /\pi r_{0}^{2} )^{1/2} \exp [ - (r/r_{0} )^{2} - 2\ln 2(t/\tau_{0} )^{2} - (i\omega_{0} t)] $$

where the pulse duration (full width at half maximum, FWHM) (*τ*_0_) is set to be 50 fs in the simulations, the 1*/e*^*2*^ beam radius (*r*_*0*_) is 5 mm, and the initial peak powers (*P*_*0*_) are respectively 100 GW, 200 GW, 400 GW and 1 TW for pulse energies of 5 mJ, 10 mJ, 20 mJ and 50 mJ. For the chirp method, the initial phase *ϕ*(*t*) is introduced by the compressor of the chirped pulse amplification (CPA) system. *ϕ*(*t*) can be expressed as $\phi (t) = 2\ln 2Ct^{2} /\tau_{c}^{2}$^25^, where *C* is the chirp value and $\tau_{c} = \tau_{0} (1 + C^{2} )^{1/2}$ is the stretched pulse duration due to *ϕ*(*t*). After introducing the initial phase *ϕ*(*t*), the electric field of the femtosecond laser pulses at the exit of the amplifier (*z* = 0) can be expressed as^25^:

2 $$ E_{1} (r,t,z = 0) = (2P_{1} /\pi r_{0}^{2} )^{1/2} \exp \{ - (r/r_{0} )^{2} - 2\ln 2(t/\tau_{c} )^{2} - i[\omega_{0} t + 2\ln 2Ct^{2} /\tau_{c}^{2} ]\} $$

where *P*_*1*_ is the peak power of the stretched pulse due to the introduction of *ϕ*(*t*).

To investigate the effect of the initial phase on the location of the starting point of the filament in the femtosecond laser filamentation^31,32^, the nonlinear wave equation (only considering the optical Kerr effect) is employed to describe the propagation dynamics of the electric field *E*_1_(*r*, *t*, *z*) of the femtosecond laser pulse:

3 $$ 2ik_{0} \frac{\partial E}{{\partial z}} + \left( {\frac{{\partial^{2} }}{{\partial x^{2} }} + \frac{{\partial^{2} }}{{\partial y^{2} }}} \right)E - k_{2} k_{0} \frac{{\partial^{2} E}}{{\partial \tau^{2} }} + 2\frac{{k_{0}^{2} }}{{n_{0} }}n_{2} IE = 0 $$

where $k_{0} = n_{0} \omega_{0} {/}c$ is the linear propagation constant with the linear refractive index $n_{0} \approx 1$ and the light speed in vacuum *c* = 3 × 10^8^ m/s. The third term on the left side of Eq. (3) describes the group velocity dispersion of the femtosecond laser pulse where *k*_2_ = 0.213 fs^2^/cm^33^ is the group velocity dispersion (GVD) coefficient of air for 800 nm, $\tau { = }t - z/v_{g}$, and *v*_*g*_ is the group velocity of the laser pulse in air. The last term on the left side of Eq. (3) represents the influence of the nonlinear refractive index where *n*_2_ = 3.9 × 10^−19^ cm^2^/W^34^ is the nonlinear coefficient of the Kerr response of air for 800 nm and *I* is the laser pulse intensity. The starting point of the filament is defined as the location where the laser intensity reaches 5 × 10^13^ W/cm^2^, i.e. the clamping intensity in the filamentation, representing the onset of a stable filament^4,35^. This simplified equation is still a nonlinear partial differential equation with high complexity, so the exact solution cannot be obtained by analytical methods. The filament distance is numerically calculated and compared with the target distance. In the simulations, a “chirp scan method”^36^ is used to vary the chirp parameter *C* continuously within a given interval. A new initial phase is generated by the chirp scan method and introduced into *E*_0_. The simulation process is repeated until the target value is reached.

### GA method

The simulation flow chart of the GA method is shown in Fig. 1b. The initial phase *φ*(*ω*) is equally divided into 128 effective phase control units in the frequency domain ranging from 762 to 864 nm, in which 16 units with a constant interspacing of 8 units are assigned values by the genetic algorithm, while the other units are assigned values by Neville’s polynomial interpolation algorithm with a maximal order of 16^37^. The phase array containing 16 control units, i.e. $X = \{ \varphi_{1} ,\varphi_{2} ,\varphi_{3} , \ldots ,\varphi_{16} \}$ are optimized by the genetic algorithm. The genetic algorithm is a heuristic search technique that incorporates the notions of the biological evolution in a computational setting. Because femtosecond laser pulse with a slowly varied frequency dependent phase can achieve the modulation of the filament location in a large distance range and meet the requirements in most practical cases, evolving all the phase elements by the genetic algorithm is unnecessary. Therefore, Neville’s interpolation method is employed in this paper, which not only speeds up the convergence of the genetic algorithm, but also induces no adverse effect on the optimization results. In Neville’s interpolation, the 16 control units are used to evaluate a polynomial using the Newton polynomial form and the divided differences recursion relation. In the GA method, the spectral phase is introduced for each spectral component of femtosecond laser pulses by SLM. Each narrow bandwidth component can be treated as a quasi-monochromatic plane wave, so any modulation phase beyond 2p can be replaced by the phase in the range of 0–2p.

**Figure 2:**
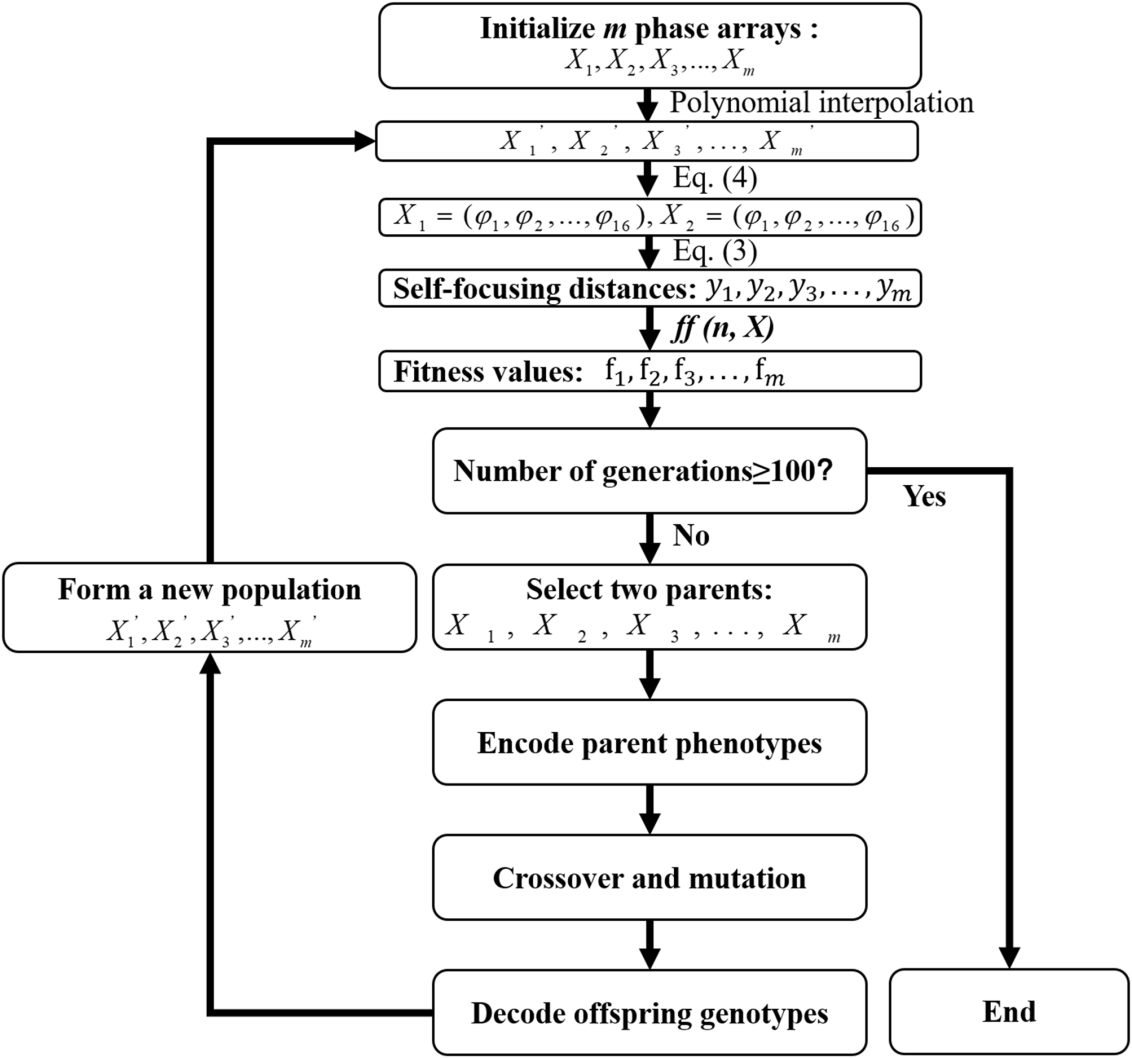
Flow chart of the genetic algorithm used to optimize the input phase *φ*(*ω*) in the frequency domain. *ff*(*n*, *X*) is the fitness value of *X* with *n* independent phase parameters.

The electric field of femtosecond laser after introducing *φ*(*ω*) can be expressed as^38^:

4 $$ E_{2} (r,t,z = 0) = F^{ - 1} \{ F\{ E_{0} (r,t,z = 0)\} \exp [i\varphi (\omega )]\} $$

where *F* and *F*^−1^ means the forward and inversed Fourier transforms respectively. The propagation dynamics of femtosecond laser *E*_*2*_ is also calculated using Eq. (3). Based on the calculated distance of the starting point of the filament, a new *φ*(*ω*) is generated by GA. The detailed flow chart of GA is presented in Fig. 2.

The GA method used to optimize *φ*(*ω*) is schematically shown in Fig. 2. Firstly, the first generation *φ*(*ω*) composed of *m* individuals (*m* = 100 in our case), i.e. $X_{1} ,X_{2} ,X_{3} , \ldots ,X_{m}$, is randomly generated. As described above, each *X* consists of *n* phase parameters, i.e. *n* “phenotypes” (*n* = 16 in our case). Then, the fitness value *ff*(*n*, *X*) of each individual is calculated using the equation $ff{ = 1/(1 + |}y - y_{0} {|})$, where *y* is the filament distance of the electric field *E*_2_(*r*, *t*, *z*) and *y*_0_ is the target value. Next, based on the calculated fitness values, two individuals *X*_*i*_ and *X*_*j*_ are selected as parents using the roulette wheel algorithm^39^. Each phenotype of the parents is converted into a sequence of 6 integers by the float-point encoding method^39^. Each integer in the sequence is termed as a gene. Therefore, the phenotypes of the parents are encoded into genotypes at this step. After that, two basic operations on genes including crossover, and mutation are implemented and offspring genotypes are generated. The crossover probability is set to be 0.85 in the simulations, which is often in the range of 0.7–0.9 for the genetic algorithm. The variation of the crossover probability in this range has little impact on the final results. The mutation mode is one-point mutation^40^ with adjustable rates based on the fitness. Through the decoding operation on the offspring genotypes, the offspring phenotypes (phase parameters) are obtained and *m* new individuals $X_{1}^{{\prime }} ,X_{2}^{{\prime }} ,X_{3}^{{\prime }} , \ldots ,X_{m}^{{\prime }}$ are produced to form a new generation. At this point, a complete iteration is finished. The above steps are repeated until the preset maximal number of generations (100 generations in our case) is reached and the individual with the fitness value most close to 1 is selected.

**Figure 3:**
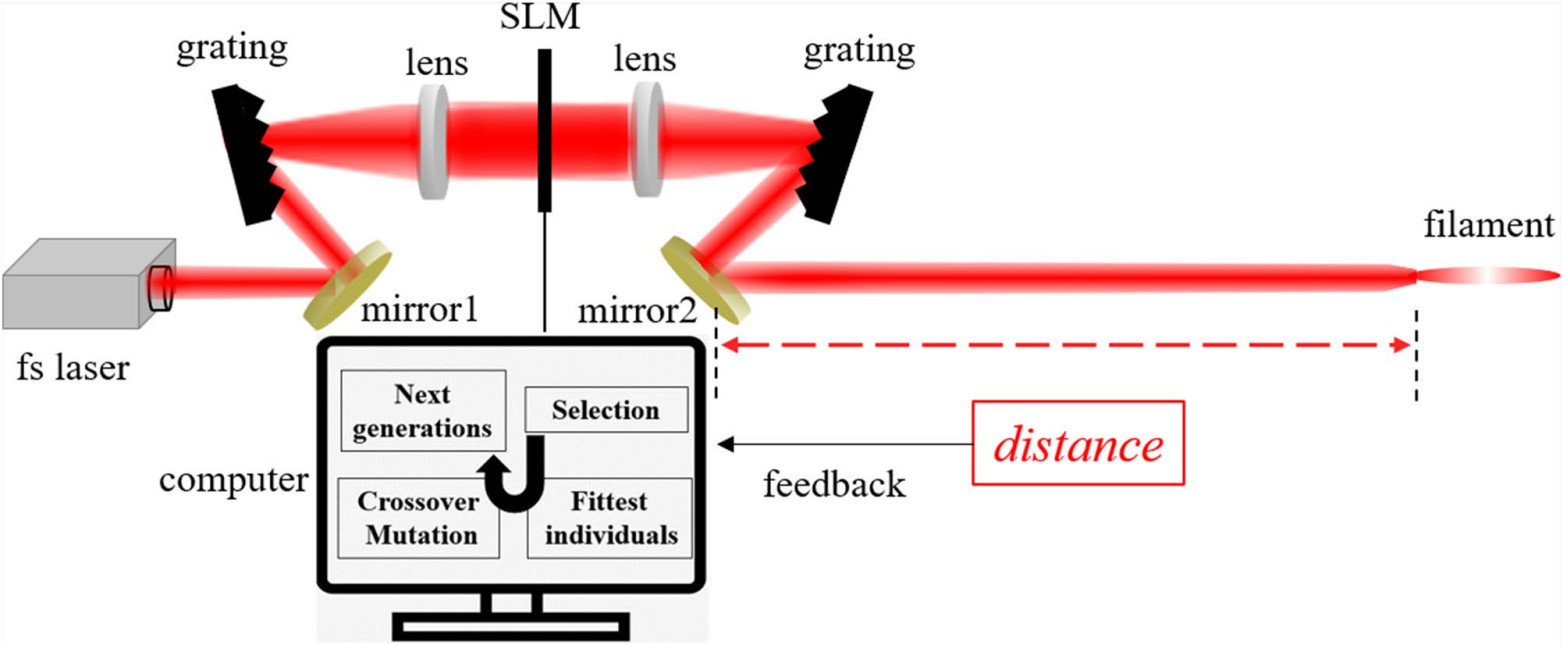
Schematic diagram of the virtual optical setup for controlling the filament location by the GA method.

To clearly show the implementation principle of this method, a virtual optical setup which can explain the realization process of the filament location control using the genetic algorithm is shown in Fig. 3. The laser pulses firstly pass through a pulse shaper which is composed of a standard 4-*f* configuration and a linear array SLM with 128 independent pixels. The phase of each pixel of SLM is controlled using the genetic algorithm and the arbitrary spectral phase can be introduced into the laser pulse. The temporal intensity profile is thus modified which affects the dynamics of femtosecond laser filamentation. The distance between the starting point of the filament and the mirror 2 is measured or calculated by the nonlinear wave equation (Eq. (3)), which is employed as the feedback for the evolution process of the genetic algorithm.

## Results and discussions

**Figure 4:**
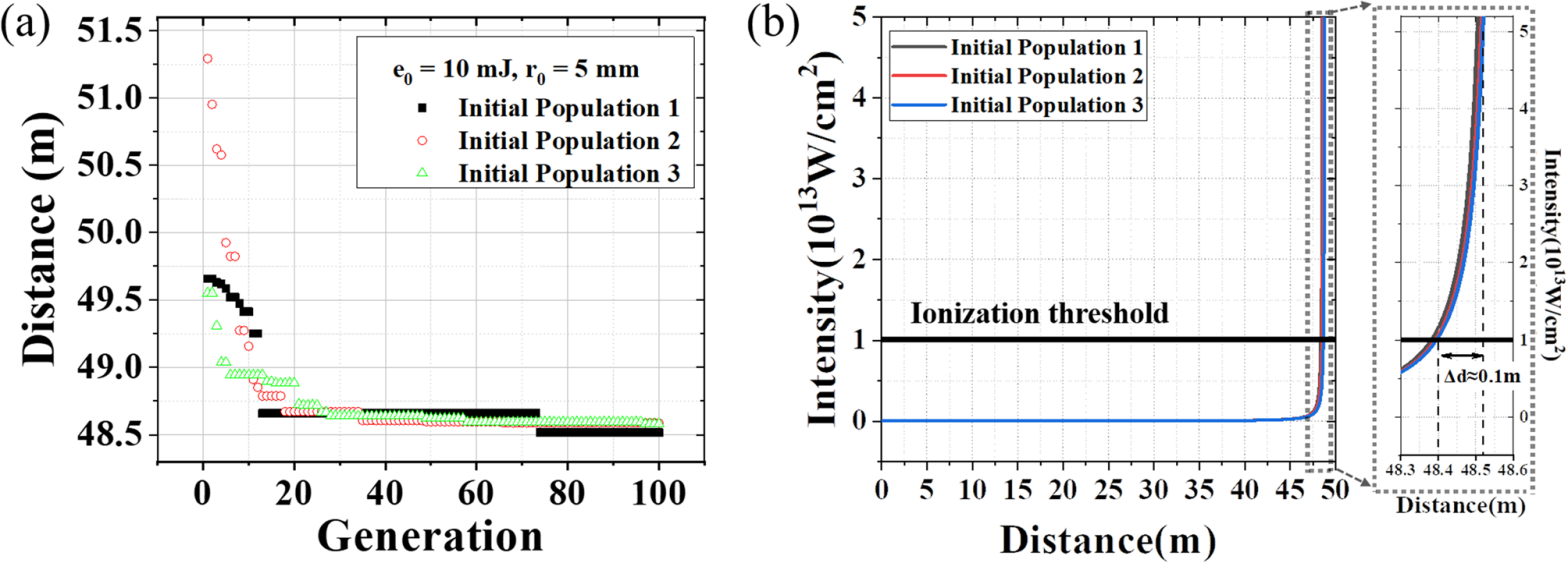
(**a**) Evolution process of finding the target filament distance (48.5 m) by the genetic algorithm. (**b**) Nonlinear propagation process of femtosecond laser pulses with the optimized spectral phases obtained in (**a**).

As shown in Fig. 4a, three different initial populations with random parameters are employed to find the target filament distance of 48.5 m using the GA adaptive control method. In Fig. 4a, e_0_ is the laser pulse energy and *r*_*0*_ is the laser beam radius (1/*e*^2^). From Fig. 4a, it can be seen that although in the initial stage, the distance values are quite different, after evolving, all the three different initial populations can reach the identical target filament distance (the fluctuations of the collapse distance are about ± 0.8% when ± 2% energy fluctuations are considered). In order to show the nonlinear collapse of the optimized laser pulses in Fig. 4a, the dependence of the laser intensity of the optimized laser pulses on the propagation distance is shown in Fig. 4b. Figure 4b presents that the catastrophic collapse happens near the target distance. Because the ionization effect is not considered in Eq. (3), the curve in Fig. 4b must be truncated when the laser intensity reaches the ionization threshold of 10^13^ W/cm^2^^41^. However, due to the catastrophic collapse, the plasma channel before the starting point of the filament is very short^42^ and thus the distance difference between the locations with the ionization threshold intensity and the clamping intensity can be negligible.

The reliability of the simulation results obtained by the GA adaptive control method is verified by comparing with the results obtained by the chirp method. For the chirp method, the frequency chirp is adjusted by changing the chirp value *C*. In the simulations, the filament location (the starting point of the filament) is investigated for femtosecond laser pulses with different pulse energies. The simulation results are shown in Table 1. The contrast test with different target distances is repeated for each pulse energy. The chirp value *C* optimized by the chirp method are listed in the last column of Table 1. It can be seen from Table 1 that the nearly identical performance in the filament control can be obtained by the chirp method and the GA adaptive method and the relative distance errors by the GA method is slightly smaller than those by the chirp method. The results shown in Table. 1 demonstrate that the distance values optimized by the GA agree well with those by the chirp method regardless of the laser pulse energy.

**Table 1:** Control of the starting point of the filament by the GA method and the chirp method when different pulse energies and target distances are used.

Pulse energy (mJ)	Target distance (m)	GA distance (m)	Relative error of GA (%)	Chirp distance (m)	Relative error of Chirp (%)	Chirp value C
5	75	74.97	0.04	77.84	3.79	− 12.2
	38.5	38.68	0.47	38.69	0.50	− 5.4
10	48.5	48.52	0.04	48.58	0.16	− 14.8
	26	26.24	0.92	26.26	1.00	− 6.2
20	33.5	33.52	0.06	33.54	0.12	− 18
	18	18.18	1.00	18.28	1.56	− 6.8
50	21	21.01	0.05	21.05	0.24	− 22.2
	11.5	11.36	1.22	11.32	1.57	− 8

In addition, the filament location control is mainly via the dispersion compensation of SLM. There are 16 phase sampling points generated by the GA method, which covers the spectrum range of 762–864 nm. Therefore, each sampling unit covers a wavelength bandwidth of 5.3 nm, i.e. a frequency bandwidth *Δv* of ~ 2.48 THz. According to the bandwidth-duration product for Gaussian laser pulses (*ΔtΔv* ≥ 0.441), the chirp-free pulse controlled by each sampling unit has a duration *Δt* of ~ 176 fs. Therefore, the dispersion length *L*_*D*_ = (*Δt* /1.66)^2^/*k*_*2*_ where *k*_*2*_ = 0.213 fs^2^/cm is the group velocity dispersion of air for 800 nm laser is calculated to be 527 m and the limitation for the distance of the collapse point can be defined by this characteristic dispersion length.

**Figure 5:**
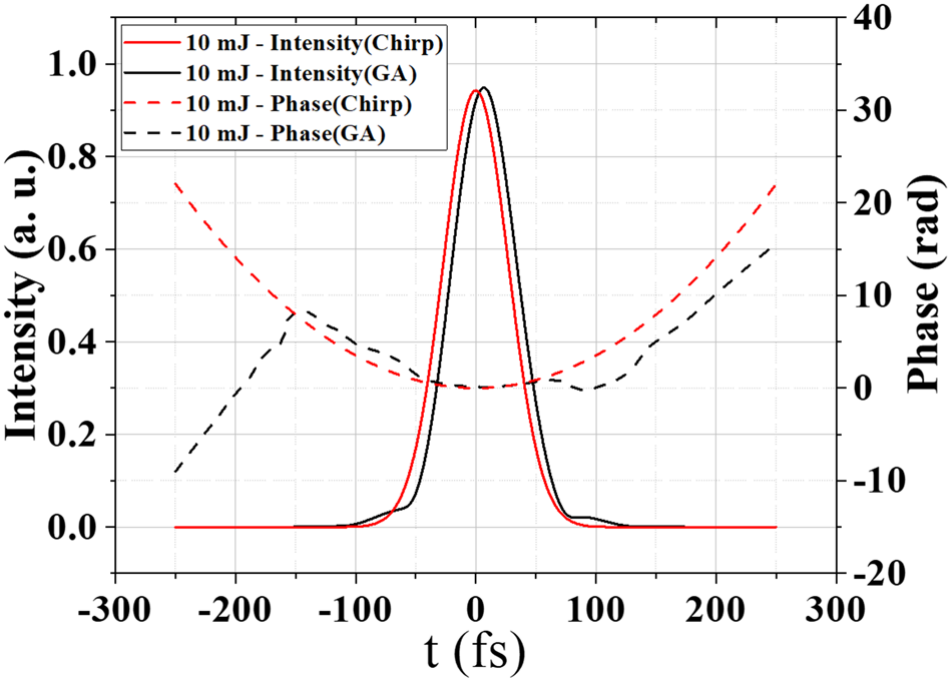
Comparison of the intensity profiles and the phase obtained by the GA method and the chirp method.

Finally, we compare the intensity profiles and the phase of 10 mJ femtosecond laser pulses with identical filamentation locations obtained by the GA and chirp methods respectively. The simulation results are shown in Fig. 5. It presents that to achieve the same filament location, the intensity profiles of the laser pulses are almost identical for the GA and chirp methods and the temporal phases obtained by these two methods are also identical within the laser pulse. It is interesting to see that over a much larger temporal window, the temporal phase obtained by the GA method exhibits a significant third-order component. It should be noted that only the phase profiles near the leading and trailing parts of the laser pulse would change when the number of iterations is over 100. However, their contributions to the location adjustment of the laser filamentation can be ignored. The higher-order phase distribution is the direct result of the more adjustable phase parameters in the GA method. This further demonstrates the feasibility and reliability of the GA adaptive control system and indicates that the GA adaptive method can achieve more complex phase modulation than the chirp method.

It is known that femtosecond laser will gain significant second-order and higher-order dispersions after propagating a long distance in air. Efficient control and elimination of high-order dispersion plays an important role in the pulse temporal profile control and laser filamentation control^43^. The chirp method always uses the grating pair to achieve the chirp modulation, resulting that the chirp method has only one adjustable parameter, i.e. the effective spacing of the grating pair. Therefore, the chirp method cannot simultaneously adjust the second-order and high-order dispersions. The genetic algorithm has much more degrees of freedom due to the employment of the phase modulation device with hundreds of pixels. Therefore, the GA method can adjust both the second-order and high-order dispersions, which is superior to the chirp method. Due to the advantages of more adjustable parameters and more flexible phase distribution that can be achieved by the GA method, the GA method can provide more precise and higher order pulse phase control and is especially useful in the practical applications in long range propagation where efficient control and elimination of high-order dispersion are required^44^.

In practice, one generation evolution by the genetic algorithm is quick enough so that the computing time can be ignored^36^. Therefore, the speed of the phase optimization for the GA method is mainly determined by the frame rate of the phase modulator used in the real apparatus. The optimization can be completed in several seconds if a high-speed SLM (High-speed 1536 SLM, BNS Inc., 1 kHz frame rate) or deformable mirror (DM292, ALPAO Inc., 2 kHz frame rate) is used.

In this study, the numerical simulations demonstrate that femtosecond laser pulse without a prior knowledge about its phase can be controlled to reach the target distance of the laser filament using the GA method. The simulation results obtained by the GA method for different pulse energies and different target distances are compared with those obtained by the chirp method, which shows a high degree of agreement. Moreover, when the target distance is fixed, the simulation-obtained intensity profile and phase of femtosecond laser pulses are identical for the two methods, which further demonstrates the feasibility of the GA method. In practice, the chirp method usually controls the filament location by tentatively introducing the chirped phase. The blind adjustment of the chirp method makes it an inefficient control method. However, for the GA method, when equipped with a fast phase modulator, it can achieve the adaptive and efficient modulation without any prior knowledge about the spectral phase.

## Data Availability

The datasets generated and analyzed during the current study are available from the corresponding author on reasonable request.
